# Clinicial-pathologic correlations of non-trauma related Odontodysplasia in 28 dogs: 2013-2023

**DOI:** 10.3389/fvets.2024.1424784

**Published:** 2024-07-08

**Authors:** Ching Ching Shirley Kot, Stephanie Goldschmidt, Natalia Vapniarsky, Boaz Arzi, Maria Soltero-Rivera

**Affiliations:** ^1^Dentistry and Oral Surgery Service, CityU Veterinary Medical Centre, Kowloon, Hong Kong SAR, China; ^2^Department of Surgical and Radiological Sciences, University of California, Davis, Davis, CA, United States; ^3^Department of Pathology, Microbiology and Immunology, School of Veterinary Medicine, University of California, Davis, Davis, CA, United States

**Keywords:** odontodysplasia, teeth, canine, endodontal disease, dentistry

## Abstract

Odontodysplasia is an uncommon dental developmental disorder associated with enamel, dentin, pulp abnormalities, and overall tooth morphology. The affected tooth is grossly abnormal in size and contour and is commonly associated with swelling of the affected area and failure of eruption. Histologically, the enamel and dentin are hypoplastic and hypomineralized. Odontodyplasia occurs most commonly in response to direct trauma to the developing tooth bud (enamel organ and dental follicle). Data on the prevalence and features of non-traumatic odontodysplasia are lacking. Medical records of dogs diagnosed with odontodysplasia were reviewed at the William R. Pritchard Veterinary Medical Teaching Hospital (VMTH), University of California, Davis, for 10 years (from 2013 to 2023). Dogs with a known history of facial trauma, persistent deciduous tooth or teeth over the region of odontodysplastic tooth or teeth, and endodontic disease of the persistent deciduous tooth or teeth were excluded from the study. Twenty-eight dogs were included in this retrospective study, representing an incidence of 1.4 per 100 dogs presenting over 10 years. Regional odontodysplasia (RO) was identified in twenty-two dogs, and generalized odontodysplasia (GO) was found in six dogs. Both comprehensive oral examination and diagnostic imaging were essential in diagnosing and assessing the presence of odontodysplasia. Awake oral examination failed to identify odontodysplasia in almost 70% of the RO cases. Secondary diseases or lesions in odontodysplastic teeth, such as periodontal disease, endodontal disease, and perio-endo lesions, were commonly seen and were particularly more frequently identified in strategic teeth (canine and carnassial teeth) than non-strategic ones. Similarities, such as female predilection, maxilla more commonly affected, and clinical signs, were observed between RO in dogs and those reported in people. The exact etiology of non-traumatic odontodysplasia remains elusive, and the condition may be of multifactorial causality.

## 1 Introduction

Odontodysplasia is an uncommon dental developmental disorder associated with enamel, dentin, pulp abnormalities, and overall tooth morphology (1). The clinical, radiographic, and histological features of odontodysplasia were first reported by Zegarelli et al. (2). The affected tooth is typically discolored, smaller, or with gross distortion of the normal anatomy of the crown (1, 2). The tooth often has an irregular surface contour with pitting and grooves (3). Most odontodysplastic teeth are intraosseously located and are unerupted (2). Histologically, enamel and dentin are hypoplastic and may be hypomineralized (1, 2). The scarceness of dental hard tissues results in reduced radiopacity of the teeth, leading to these being described as “ghost teeth” (1).

Odontodysplasia may be classified into regional (RO) and generalized odontodysplasia (GO). The term “regional” was adopted following Zegarelli et al.’s description of the condition affecting several adjacent teeth in a particular jaw segment. The number of teeth and quadrants involved with RO varies (1). Some authors consider RO, which affects three or more quadrants, as GO (3); some describe the condition as GO when all quadrants of the oral cavity are affected (4–6). RO in humans is not hereditary (7), and the pathogenesis is unknown (3). It occurs more frequently in maxillary teeth than mandibular teeth and deciduous and permanent dentition may be affected (7). The most common presenting findings of odontodysplasia are failure of tooth eruption and associated swelling of the affected area (3). In this study, we describe the two forms of odontodysplasia (regional vs. generalized) based on the anatomical distribution of the odontodysplastic teeth.

Human medical literature on odontodysplasia is mainly limited to case reports, and there are a total of 161 RO cases published in English between 1953 and 2017 (3). In contrast, the veterinary literature describes dental conditions resembling odontodysplasia in various species, including dogs (8–14), cats (15, 16), horse (17), domestic ferrets (18), and several wildlife animals (19–21). Despite several factors that have been suggested to correlate with the anomaly, most odontodysplasia cases, especially RO, shared a common history of prior local trauma. Non-trauma-related odontodysplasia, therefore, appears to be understudied. There is only one article describing RO in a juvenile dog without any known history of facial trauma, persistent deciduous tooth, or endodontic disease of the deciduous tooth over the area of anomaly (9). Another article described generalized enamel, dentin, and root hypoplasia consistent with GO in an 18-month-old dog with unknown prior history (8). The present descriptive study aims to evaluate the incidence, clinical presentation, diagnosis, and treatment of non-trauma-related odontodysplasia in dogs and the secondary disease or lesion associated with this rare anomaly.

## 2 Materials and methods

The medical records of dogs diagnosed with odontodysplasia in William R. Pritchard Veterinary Medical Teaching Hospital (VMTH), University of California, Davis, U.S.A. (2013–2023) were identified and assessed. Dogs with a known history of facial trauma, persistent deciduous teeth or teeth over the region of odontodysplastic tooth or teeth, and endodontic disease of the persistent deciduous tooth or teeth were excluded from the study. Data retrieved from the medical records included signalment, reason for clinical presentation, history of co-morbidities, previous dental treatment, travel history, and oral examination findings. Specific oral findings that were documented included occlusion, as defined by the American Veterinary Dental College (22), which skeletal malocclusion includes class 2, 3, and 4 malocclusions, but not class 1 malocclusion, site, and tooth type affected by odontodysplasia, tooth eruption status, as well as periodontal and endodontal status of the odontodysplastic tooth. Due to their critical functional properties, canine and carnassial teeth (maxillary fourth premolar and mandibular first molar teeth) were classified as strategic teeth. The diagnostic imaging modality (computed tomography [CT], cone-beam computed tomography [CBCT], dental radiography) used for the identification of odontodysplasia was noted. Results of histopathology, when available, were recorded. Lastly, treatment and its rationale were collated.

Regional odontodysplasia was diagnosed when one or more teeth in a quadrant or site were affected, with a maximum of three quadrants involved. Generalized odontodysplasia was diagnosed when all four quadrants or sites were affected as previously described (4–6). The awake oral examination findings were compared with the anesthetized oral examination findings (dental charting) and diagnostic imaging to determine if odontodysplasia was incidental. Incidental finding was defined as “an incidentally discovered mass or lesion, detected by CT, MRI, or other imaging modality performed for an unrelated reason” (23).

Descriptive data were calculated. Normally distributed variables were expressed as mean (standard deviation, SD), and those not normally distributed were expressed as median (range). Categorical data were expressed as frequencies and percentages. For RO cases, the data were also stratified by sex, breed, occlusion, and tooth type to assist in comparing the prevalence and site of occurrence of RO across various groups. Fisher’s exact test evaluated the relationship between groups (sex, breed, and occlusion) and the number of RO-affected teeth. The Cochran–Mantel–Haenszel test (CMH) was used to investigate the relationship between groups (dental arch type and tooth type) and the presence of RO tooth. The CMH test was also used to assess the relationship of sex, breed, occlusion, dental arch type, or tooth type with secondary disease or lesion associated with odontodysplasia. For all analyses, *p* < 0.05 was considered statistically significant.

## 3 Results

Between 2013 and 2023, 28 dogs with non-trauma-related odontodysplasia were identified out of the 2,012 dogs with comprehensive oral examinations and a complete set of full-mouth intraoral radiographs or analogous imaging performed. Consequently, the incidence was 1.4 per 100 dogs during the reported period. Twenty-two dogs were affected by RO, and six dogs were affected by GO.

### 3.1 Regional odontodysplasia

Forty-one affected teeth with RO were identified in twenty-two dogs over 10 years. Nine dogs (40.9%) had only one tooth affected by RO, seven dogs (31.8%) had two affected teeth, and six dogs (27.3%) had three teeth affected. No dog had more than three teeth affected by RO. The median number of RO-affected teeth per dog was 2 (Table 1).

**Table 1:** Clinical data of regional odontodysplasia (RO) cases.

Clinical data of regional odontodysplasia (RO)	Number of dog (%)
*Occlusion*	
Normal occlusion	11 (50)
Class 1 malocclusion	4 (18.2)
Class 2 malocclusion	2 (9.1)
Class 3 malocclusion	3 (13.6)
Class 4 malocclusion	1 (4.5)
Not assessed	1 (4.5)
*Jaw*	
Maxilla only	11 (50)
Mandible(s) only	7 (31.8)
Both	4 (18.2)
*Number of sites (quadrants) involved*	
One single	14 (63.6)
Two	6 (27.3)
Three	2 (9.1)
*Number of teeth involved*	
One single	9 (40.9)
Two	7 (31.8)
Three	6 (27.3)
More than three	0 (0)

#### 3.1.1 Patient clinical presentation

The mean age (SD) of the dogs with RO was 4.19 years (3.71), ranging from 5 months to 12 years. Eighteen (81.8%) were pure-breed dogs, while four (18.2%) were mixed-breed dogs. Among the pure-breed dogs with RO, Labrador retriever was the most commonly identified breed (*n* = 4), followed by German shepherd dog (*n* = 2), rottweiler (*n* = 2), and chihuahua (*n* = 2), Chinese crested dog (*n* = 1), Havanese (*n* = 1), pug (*n* = 1), pomeranian (*n* = 1), Boston terrier (*n* = 1), golden retriever (*n* = 1), dachshund (*n* = 1), and miniature poodle (*n* = 1). Fifteen females (68.2%, 13 spayed and two intact) and seven males (31.8%, six neutered and one intact) were diagnosed with RO. The female-to-male ratio of the affected dogs was 2.14:1. Both breed (pure vs. mixed) and sex (male vs. female) showed no significant difference in the number of RO-affected teeth (*p* = 0.64, *p* = 0.73, respectively).

Eighteen dogs (81.8%) did not have reported co-morbidities; four dogs (18.2%) were reported to have the following co-morbidities: myxomatous mitral valve disease, and intervertebral disc disease (*n* = 1), chronic acetabular fracture (*n* = 1), mast cell tumor (*n* = 1), and seizure with historic canine distemper infection (*n* = 1). None of the dogs had received prior dental treatments, and all had lived with their owners in California since they were acquired.

Eight dogs (36.4%) were initially presented for evaluation of periodontal disease. Other presenting complaints were malocclusion (*n* = 3), tooth fracture (*n* = 2), tooth discoloration (*n* = 2), suspected dentigerous cyst (*n* = 2), unerupted tooth (*n* = 1), dental abscess (*n* = 1), dental anomaly (*n* = 1), intraoral draining tract (*n* = 1), persistent deciduous tooth (*n* = 1), and palatal mass (*n* = 1). The presenting complaints of 10 dogs (45.5%) were later deemed related to the secondary disease or lesion associated with RO.

#### 3.1.2 Oral findings

Collectively, fifteen dogs had normal occlusion and jaw length relationships, while six dogs had skeletal malocclusion (Table 1). There was no significant difference between the number of RO-affected teeth in dogs in these two groups (*p* = 0.33).

**Figure 1:**
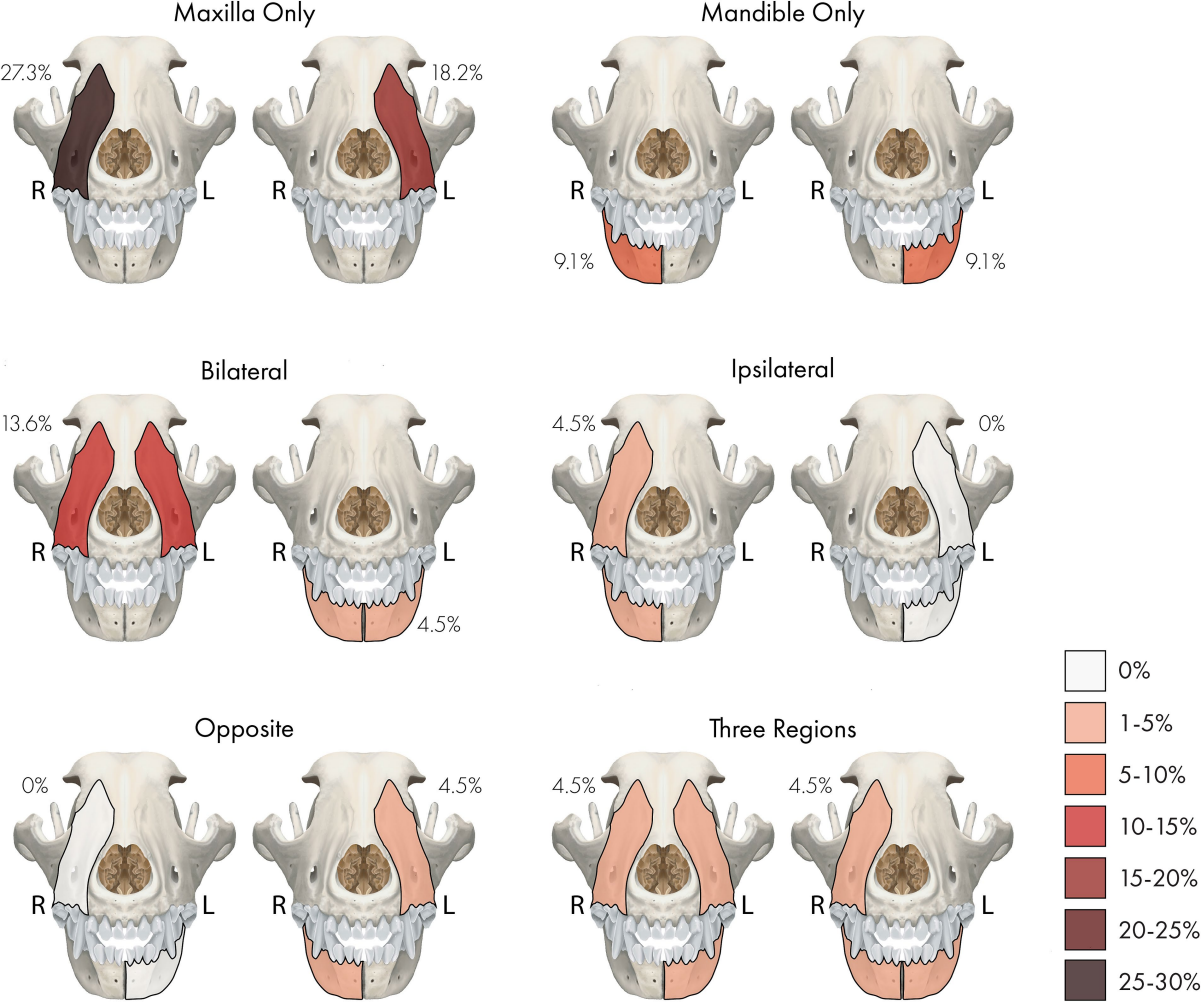
A percentage heatmap showing the distribution and number of sites/regions involved with regional odontodysplasia (RO). Percentages represent the percentage of dogs with RO-affected tooth or teeth in that site or region. The letter denotes the side as follows: R = right, L = left.

The right maxilla was the most commonly affected site, followed by the left maxilla (Figure 1). The majority, 25 teeth (61% of affected teeth), were in the maxilla, and 16 teeth (39%) were in the mandible (Table 2). When taking all 875 teeth available in 22 dogs for evaluation into account, the maxillary and mandibular arch showed no significant difference in the presence of an RO tooth (*p* = 0.11). All of the RO-affected teeth were permanent, except in one dog, in which a deciduous tooth and a permanent tooth were affected by RO. Most affected teeth (70.7%) erupted fully (Table 2).

**Table 2:** Clinical data of teeth affected by regional odontodysplasia (RO).

Clinical data of regional odontodysplasia (RO)	Number of RO-affected teeth (%)
*Location of the RO teeth*	
Maxilla	25 (61)
Mandible(s)	16 (39)
*Eruption status*	
Fully erupted	29 (70.7)
Partially erupted	4 (9.8)
Unerupted	8 (19.5)
*Tooth type*	
Incisor tooth	8 (19.5)
Canine tooth	6 (14.6)
Premolar tooth	14 (34.1)
Molar tooth	13 (31.7)

**Figure 2:**
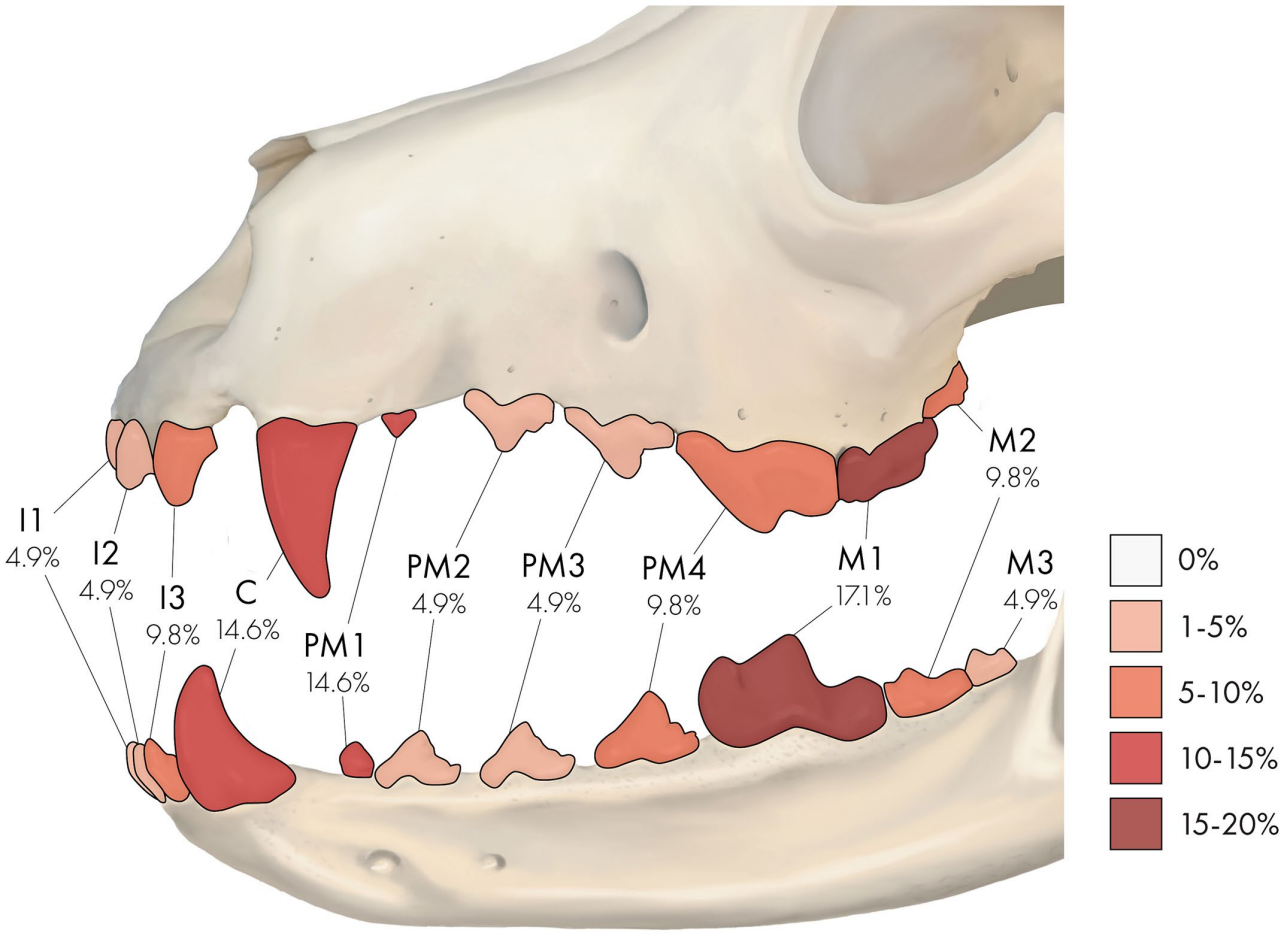
A percentage heatmap displaying the portion of specific tooth types affected by regional odontodysplasia (RO). Percentages represent the percentage of tooth type affected by RO. The letter denotes tooth type as follows: I = incisor tooth, C = canine tooth, PM = premolar tooth, M = molar tooth. The number after the letter denotes a specific tooth as follows: 1 = first, 2 = second, 3 = third, and 4 = fourth. For example, 9.8% at PM4 means that 9.8% of the RO-affected teeth were fourth premolar teeth.

Collectively, 14 RO-affected teeth (34.1%) were strategic, while 27 (65.9%) were non-strategic teeth (Supplementary Table S1). Specific tooth types and teeth affected by RO are illustrated in Table 2 and Figure 2. There was no significant difference between the presence of RO teeth in various tooth types (*p* = 0.25). However, RO was significantly more commonly seen in strategic teeth than non-strategic teeth (*p* = 0.02). When considering the affected site, the right and left maxillary canine teeth and the right mandibular first molar tooth were the most frequently identified teeth with RO (each accounted for 7.3% of the total affected teeth). These results are comprehensively presented in Supplementary Table S1.

Of the seven dogs with two RO-affected teeth, three (42.9%) had bilateral involvement of the same tooth type, two (28.6%) had the affected teeth adjacent to each other, and two (28.6%) had the affected teeth seen in contralateral sites of the opposite jaw (i.e., left maxilla and right mandible). Conversely, among the six dogs with three teeth affected by RO, half (50%) had the affected teeth at the same site and adjacent to each other.

**Figure 3:**
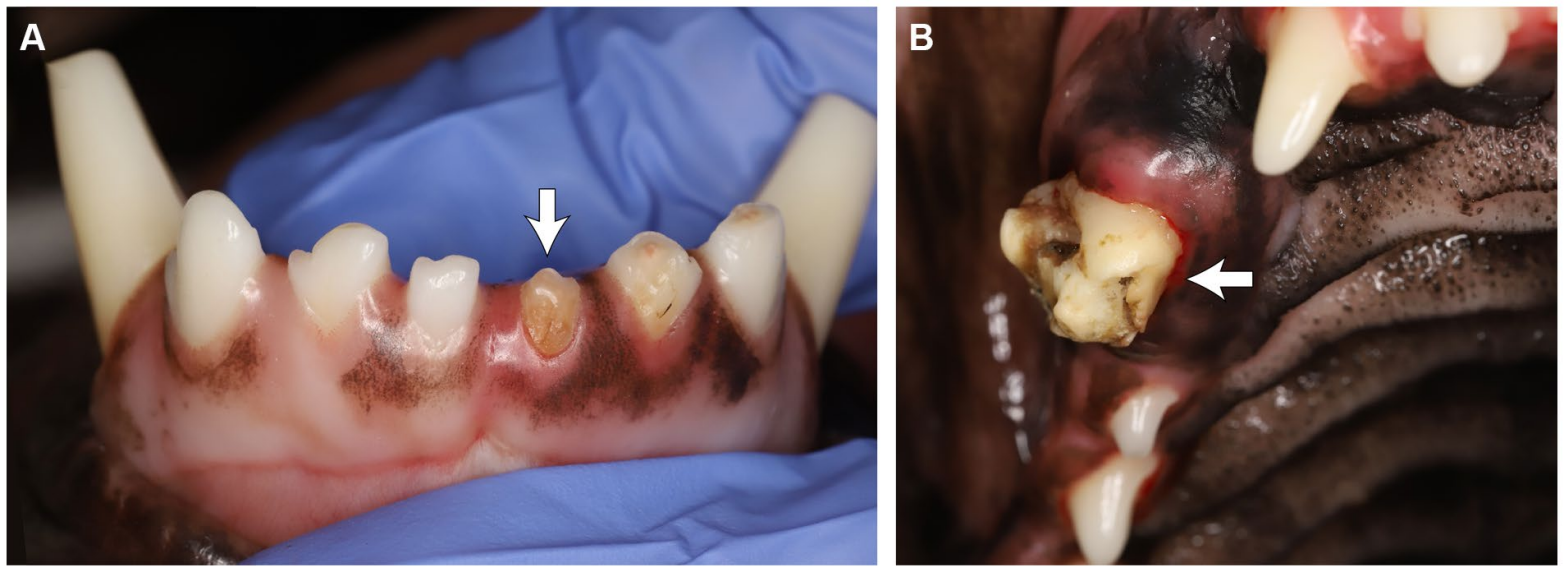
**(A)** Odontodysplastic left mandibular first incisor tooth. **(B)** Odontodysplastic right maxillary canine tooth. Note the tooth discoloration, irregular contour and surface, and pits and grooves.

Seven of the twenty-two RO cases (31.8%) had reported odontodysplasia on at least one site in the awake oral examination, while the remaining fifteen cases (68.2%) did not. The RO condition reported in those 15 cases was identified via anesthetized oral exam and diagnostic imaging. Anesthetized oral examination findings described the RO-affected teeth as discolored (yellow or yellowish-brown), irregularly contoured with a rough surface and the presence of pits and grooves, and appeared to be hypoplastic (Figure 3).

#### 3.1.3 Diagnostic imaging findings

**Figure 4:**
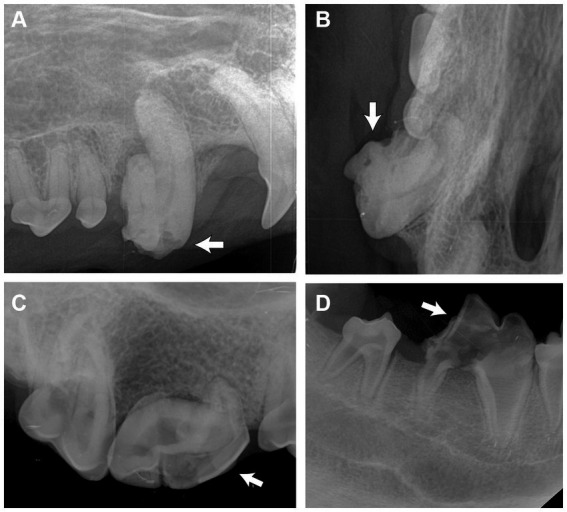
Intraoral radiographs of odontodysplastic strategic teeth (arrow). **(A)** The right maxillary canine tooth was malformed with an abnormal crown shape and short root, as shown in the lateral and **(B)** occlusal views. **(C)** The crown and root of the right maxillary fourth premolar tooth were malformed. A relatively larger pulp cavity was seen. **(D)** Reduced radiodensity of the crown and root of the right mandibular first molar tooth.

Diagnostic imaging was performed in all cases. One single imaging modality was used in nineteen cases (86.4%), and two were used in three cases (13.6%). Among the three reported diagnostics adopted, intraoral dental radiography was most commonly used (72%), followed by CBCT (20%) and conventional CT (8%). Radiologically, the affected teeth were abnormally shaped and malformed, with reduced radiodensity of the crown. The roots of some of the affected teeth were absent or hypoplastic; some of them were irregularly shaped. The enamel and dentin were thin and might not have a clear demarcation between the two layers (Figure 4). The pulp cavity anatomy of RO teeth was subjectively better visualized in CT and CBCT, in which some pulp cavities appeared obliterated while some appeared widened. RO was listed as an incidental finding in the medical records in five cases (22.7% of the total RO cases), in which eight unerupted RO teeth (19.5% of the total number of RO-affected teeth) were identified via diagnostic imaging.

#### 3.1.4 Secondary lesions

Secondary disease and lesions were identified in thirty-two of the RO-affected teeth (78.0%), whereas the remaining nine (22.0%) showed the absence of clinical or radiographic signs of secondary lesions. Among the 32 teeth, 28.1% had endodontal disease, with the majority assessed as periapical lesions (i.e., increased periapical lucency in diagnostic imaging), followed by complicated crown fracture and pulpal necrosis; 18.8% had period-endo lesions; 18.8% had the severe periodontal disease; 18.8% had moderate periodontal disease, and 15.6% were associated with either rhinitis (deemed related to an unerupted odontodysplastic tooth in the nasal cavity) or a cyst. No significant difference was observed regarding the presence of secondary disease in RO teeth between the breeds (pure vs. mixed), sex (male vs. female), two arches (maxillary vs. mandibular), or tooth types (*p* = 1, *p* = 0.25, *p* = 0.29, *p* = 0.31, respectively). Significantly more RO teeth with secondary disease were seen in dogs with normal jaw length relationships than those with skeletal malocclusion (*p* = 0.01) and in strategic teeth than non-strategic teeth (*p* = 0.01).

#### 3.1.5 Histology (*n* = 5)

**Figure 5:**
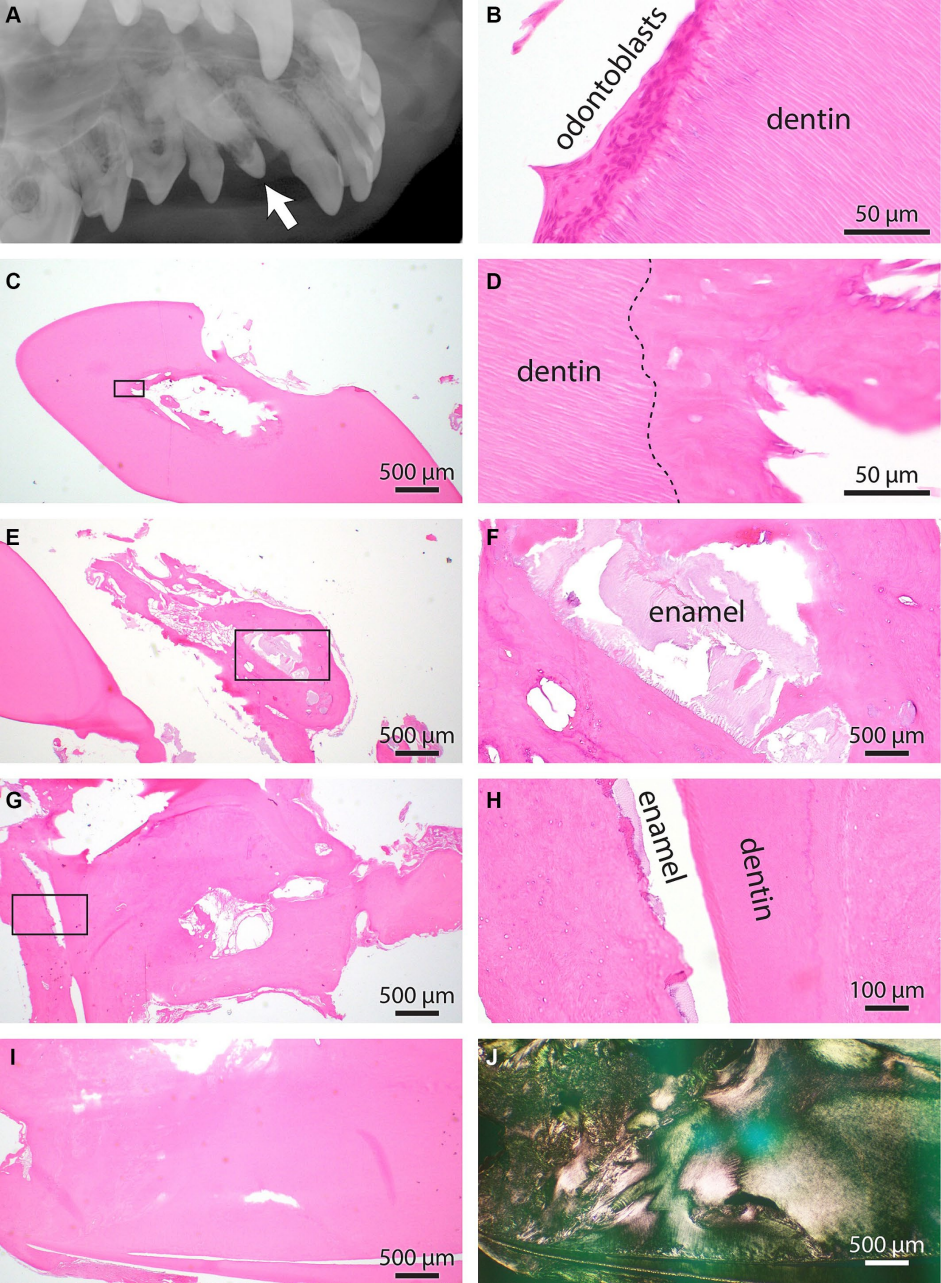
**(A)** Intraoral radiograph of the unerupted odontodysplastic right maxillary canine tooth (arrow) and its adjacent teeth, lateral view. **(B)** Normal pulp and dentin interface histology from the right maxillary canine tooth. Note the unique tubular arrangement of dental matrix fibers intimately interfacing the layer of odontoblasts. **(C)** Low magnification of the right maxillary first premolar tooth. A cemento-osseous matrix with no apparent osteoblast or other pulp cavity elements obliterates the pulp cavity. **(D)** Higher magnification of the rectangle-enclosed area in C. The interrupted line denoted the dentin interface with cemento-osseous matrix obliterating the pulp cavity. **(E)** Low magnification of right maxillary canine tooth at the root aspect. An excessive cement matrix with islands of pale basophilic material is enclosed. **(F)** Higher magnification of the rectangle-enclosed area in E. The histomorphology of basophilic material is consistent with the enamel matrix. **(G)** Additional information is from the apical aspect of the right maxillary canine tooth. The dental matrix is split into two pillars surrounded by a cemento-osseous matrix. The cemento-osseous matrix is cavitated and has irregular bays, islands, and whorls. **(H)** Higher magnification of the rectangle-enclosed area in G. Note a thin slit between dentin and cemento-osseous matrix positioned on each side. The slit space contains remnants of enamel matrix. **(I)** An additional area of the right maxillary canine tooth’s apical aspect demonstrates abnormally interwoven dentin and cemento-osseous matrixes. **(J)** The section depicted in I was imaged under polarized light to underscore the abnormal orientation of dental and cemento-osseous matric fibers.

**Figure 6:**
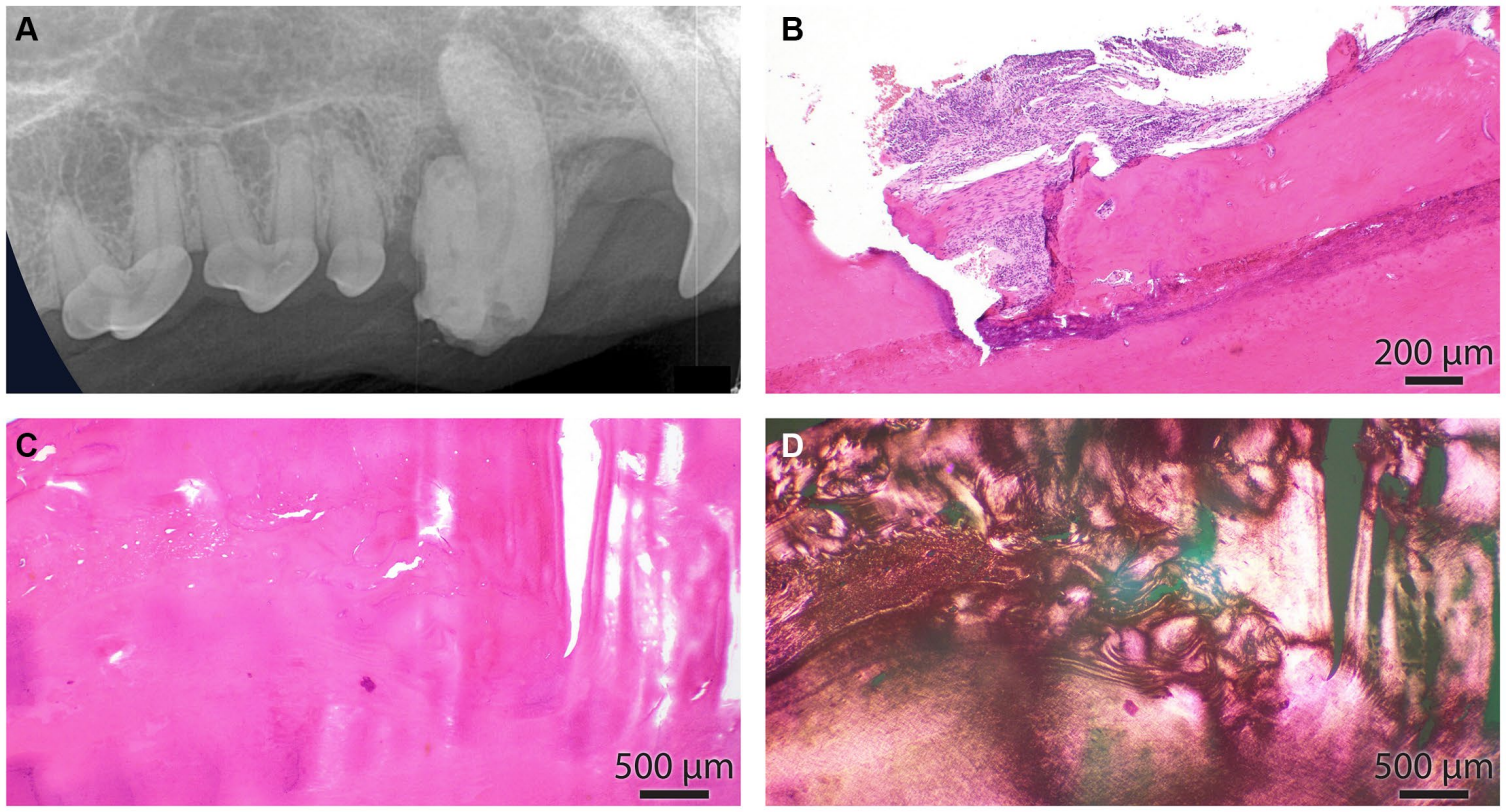
**(A)** The intraoral radiograph of the odontodyspolastic right maxillary canine tooth, lateral view, is the same as in Figure 4A. **(B)** The cemento-dental junction of the canine tooth is depicted in A. The cementum is focally resorbed by periodontal inflammation. **(C)** Dental and cemento-osseous matrixes are interlacing in an irregular pattern. **(D)** Section depicted in C under polarized light. This image underscores the haphazardous arrangement of collagen fibers in dental and cemento-osseous matrixes.

Histologically, uniform features were characterized by irregular islands and bays of dentin interlacing with cemento-osseous and possibly enamel matrixes. The interface between these matrixes was scalloped and ragged. Occasional small islands of presumed enamel matrix were embedded within the cemento-osseous matrix or between the dentin and cementum matrix. In one case, the amount of cemento-osseous matrix was exuberant, interpreted as hypercementosis (Figures 5, 6).

Due to the abnormal shape of the teeth, the pulp cavities were only segmentally apparent in histologic sections. In either of the examined pulp cavities in odontodysplastic teeth, odontoblasts were identified, and no other viable pulp elements were seen. Sometimes, the pulp cavity was lined by a cemento-osseous matrix or obliterated by round islands of the cemento-osseous or dentin-like matrix (presumed pulp stones). However, it was impossible to determine if this matrix was ossified *in situ* because all the histological samples underwent a decalcification process. In other instances, there was evidence of lymphoplasmacytic and neutrophilic pulpitis and periapical inflammation.

#### 3.1.6 Treatment and follow-up

**Figure 7:**
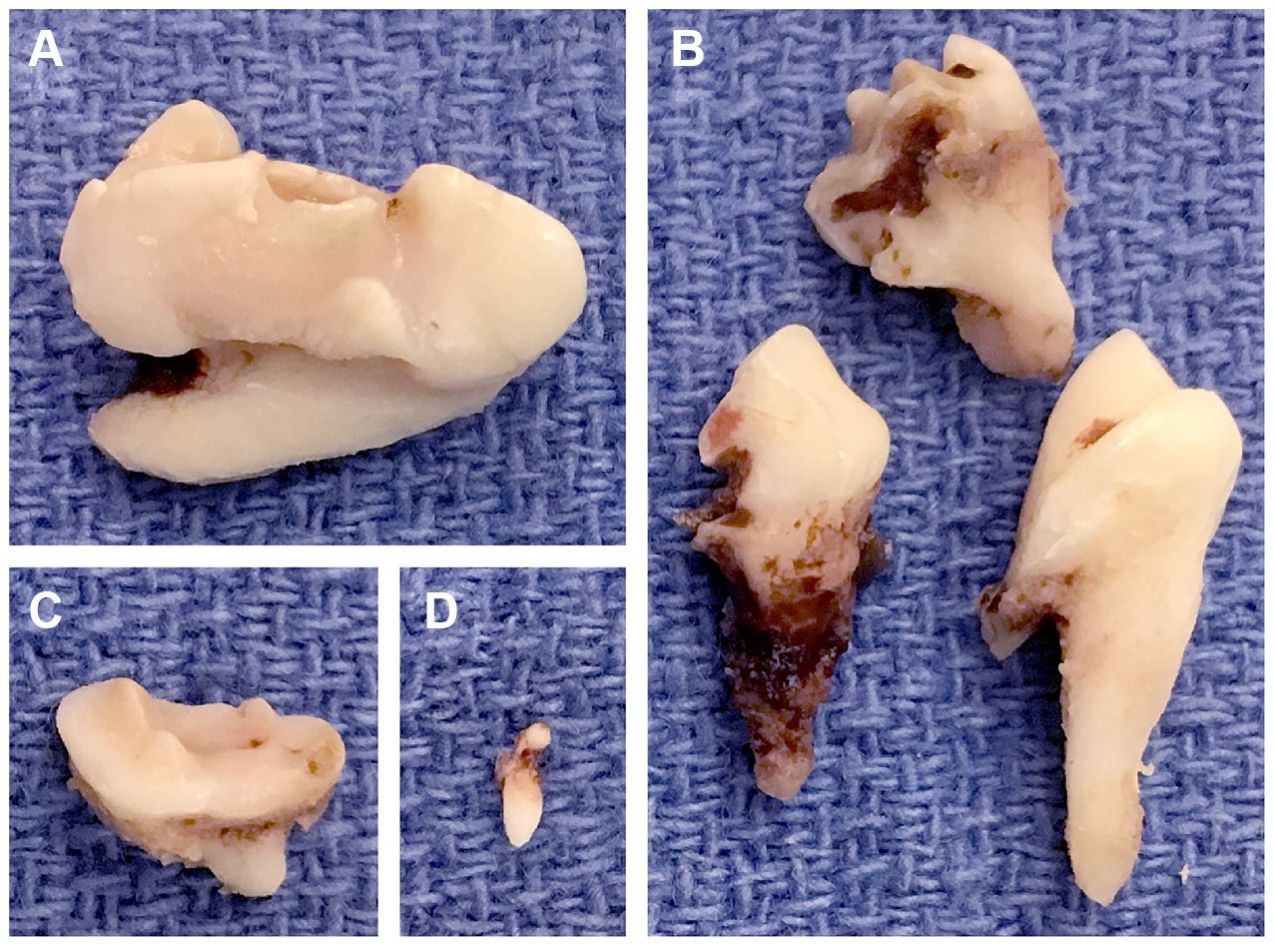
Extracted odontodysplastic teeth. **(A)** The right maxillary fourth premolar tooth. **(B)** The right maxillary first molar tooth that was sectioned. **(C)** The right maxillary second molar tooth. **(D)** The rudimentary tooth, a supernumerary tooth found adjacent to the right maxillary fourth premolar tooth.

All the teeth with evidence of secondary disease were extracted (Figure 7), whereas those without were not extracted. Continuous monitoring of the RO-affected teeth and annual oral examination were advised for the latter.

Of the 17 dogs with odontodysplastic teeth extracted, 14 (82.4%) returned for a follow-up. The follow-up period ranged from 11 to 1,116 days with a mean (SD) of 146.4 days (337.6). All extraction sites were deemed clinically healed in the awake oral examination, except one assessed as wound dehiscence and eventually healed in 2 weeks. Of the five dogs that did not have odontodysplastic teeth extracted, two (40%) returned for a follow-up. The follow-up period was 379 and 384 days, with a mean of 381.5 days. One dog developed severe periodontal disease in the odontodysplastic tooth, as evident in the comprehensive oral examination and radiographic assessment. The tooth was thus extracted. In another dog, the unerupted odontodysplastic teeth showed no radiographic changes at the follow-up appointment compared to the initial assessment. These teeth were not extracted; continuous monitoring and annual oral examination were recommended.

### 3.2 Generalized odontodysplasia

One hundred and seventy-five teeth were identified as affected by odontodysplasia in six dogs. The entire dentition was reported to be affected, except in one dog whose left and right third mandibular molar teeth were deemed normal.

#### 3.2.1 Patient clinical presentation

The mean age (SD) of the dogs with GO was 3.67 years (2.66), ranging between 2 to 8 years. Out of the six dogs affected by GO, pure-breed and mixed-breed dogs were equally represented. The three pure-breed dogs identified were a golden retriever, a rottweiler, and a Labrador retriever dogs. Five females (83.3%, all spayed) and one male (16.7%, neutered) were assessed as GO. The female-to-male ratio of the affected dogs was 5:1. Two dogs (33.3%) were presented for evaluation of periodontal disease; the presenting complaints in other dogs were tooth discoloration (*n* = 1), pain on opening mouth (*n* = 1), oral squamous cell carcinoma (*n* = 1), oral papilloma (*n* = 1). Four dogs (66.7%) had a known history of canine distemper infection as puppies; one had persistent neurological signs. None of the dogs had received prior dental treatments, and they had all lived with their owners in California since they were acquired. One dog was reported adopted from Texas.

#### 3.2.2 Oral findings

Occlusion was assessed in five dogs. Normal occlusion was reported in two dogs (40%); Class 1 malocclusion was seen in one dog (20%), and Class 3 malocclusion was identified in two dogs (40%). GO was seen in permanent dentition of all six dogs. All six dogs had multiple presumably congenitally missing teeth except one. Most of the one hundred and seventy-two teeth (98.3%) were fully erupted, one tooth (0.6%) was unerupted, and two teeth (1.1%) were partially erupted.

**Figure 8:**
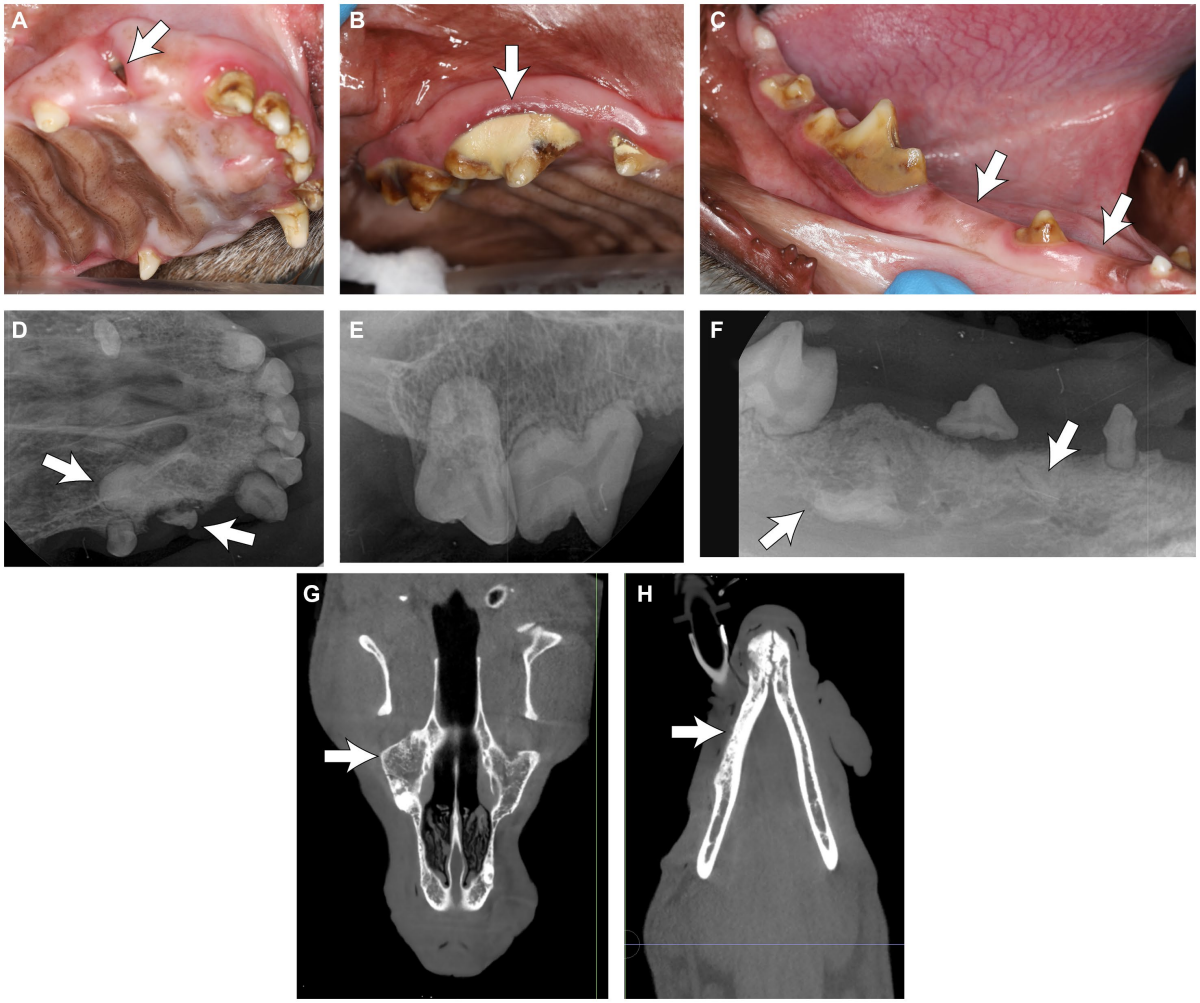
**(A–C)** Clinical photos of the rostral teeth in a dog with generalized odontodysplasia. **(A)** Note the partially erupted tooth at the right rostral maxillary region (arrow). **(B)** The odontodysplastic right maxillary premolar teeth and molar tooth were discolored. Note the heavy calculus deposition on the tooth crown (arrow). **(C)** The odontodysplastic right caudal mandibular teeth had abnormal crown shape and were discolored. The right mandibular second and fourth premolar teeth were clinically missing (arrow). **(D–F)** Corresponding intraoral radiographs of the region are shown in **(A–C)**. **(D)** The right maxillary canine tooth was malformed with an amorphous root structure (arrow). **(E,F)** The roots were hypoplastic or absent. **(F)** Some elements of tooth density were embedded in bones (arrows), resembling unerupted odontodysplastic premolar teeth. **(G)** The coronal plane of the skull CBCT of the maxilla at the level of the midface and **(H)** the mandible at the level of the mandibular canal in the same dog, with a decreased trabecular density of the skull bones with trabecular sclerosis (arrows).

Five of the six GO cases (83.3%) reported odontodysplasia during the awake oral examination, while the remaining one (16.7%) did not. The following terms were used to describe the affected teeth: “discolored,” “small,” and “enamel hypoplasia” (Figures 8A–C). All dogs underwent an anesthetized oral exam and further diagnostics.

#### 3.2.3 Diagnostic imaging findings

One imaging modality was used in three cases (50%), and two were used in three cases (50%). Among the three reported diagnostics adopted, intraoral dental radiography was most commonly utilized (62.5%), followed by conventional CT (25%) and CBCT (12.5%). Radiologically, the affected teeth were small and malformed; their crown and root had reduced radiodensity; the enamel and dentin might not have a clear demarcation between the two layers, similar to RO. The roots were short or absent, and the pulp was relatively enlarged. Some elements of tooth density were embedded in bones, resembling unerupted odontodysplastic teeth or teeth (Figures 8D–F). Incidentally, the CBCT of one dog showed decreased trabecular density of the skull bones with trabecular sclerosis (Figures 8G,H) diffusely. Subjectively, there was no difference in the appearance of RO and GO-affected teeth.

#### 3.2.4 Secondary lesions, treatment, and follow-up

Secondary disease and lesions were identified in 66 of the affected teeth (37.7%). They were extracted primarily due to the presence of periodontal disease (90.9% of the extracted teeth), with the reported assessment as mobility grade 3 and furcation grade 3; endodontal disease (7.6%) with an assessment of either root fracture or loss of vitality; and association with an intraoral fistula (1.5%). Histopathology of the affected tooth was not performed in any of the cases. Follow-up data was available in three dogs. The follow-up period ranged from 11 to 17 days, with a mean (SD) of 15 days (3.46). All extraction sites healed uneventfully, as noted by awake oral examination.

## 4 Discussion

This retrospective study is the first to collectively describe the clinical, radiological, and histological features of odontodysplasia in dogs with an unknown history of maxillofacial trauma. Despite being the largest and most comprehensive study, the etiology of non-trauma-related odontodysplasia could not be deduced and remains idiopathic. Our results demonstrated the importance of comprehensive oral exams and adopting diagnostic imaging in diagnosing and assessing odontodysplasia, as the latter helped uncover RO in more than 20% of the cases studied. Secondary diseases or lesions in odontodysplastic teeth were commonly seen and were more frequently identified in strategic teeth than non-strategic ones.

Clinically, odontodysplastic teeth in dogs are discolored, abnormally shaped, relatively small, and exhibit abnormal enamel and dentinal defects. However, awake oral examination failed to identify odontodysplasia in almost 70% of the RO cases in our study, signifying the limitations of awake oral examination in diagnosing and assessing the anomaly. Although we showed that RO-affected teeth were more commonly seen in strategic teeth, which are relatively larger and easily visualized than other teeth in the oral cavity, a portion of the affected teeth were located far caudal, either being unerupted or partially erupted and had evidence of periodontal disease which might have moderate to heavy deposition of plaque and calculus on the tooth crown, resulting in difficulty to visualize the odontodysplasia. Anesthetized oral examination of dogs in our study allowed comprehensive assessment of fully erupted teeth in a controlled setting, in which their clinical periodontal and endodontal status were documented. However, it cannot assess unerupted, impacted, or partially erupted teeth and other pathologies associated with radicular and periapical areas.

Since almost one-fifth of the odontodysplastic teeth in RO cases reported in our study were unerupted, diagnostic imaging plays an invaluable role, as imaging helped uncover 22.7% of the total RO cases. Besides enabling the detection of unerupted or impacted odontodysplasitc teeth, diagnostic imaging allows assessment of morphology of the affected tooth and recognition of possible pathology associated with odontodysplasia, namely periodontal disease featuring presence and extent of bone loss and furcation involvement or exposure, endodontic disease featuring presence of periapical radiolucency and failure of narrowing of pulp, perio-endo lesion, and cyst, as illustrated in the study. It is also worth noting that there was only one RO case with the presenting complaint listed as a “dental anomaly” (referring directly to the RO-affected tooth). In contrast, the remaining 21 RO cases were presented or referred in for reasons other than odontodysplasia. After evaluating the anesthetized oral examination and radiological findings, the presenting complaints of 10 dogs (45.5%) were later deemed related to the secondary disease or lesion associated with RO. The data highlights the possibility of a dental anomaly going unnoticed and the importance of performing a comprehensive oral examination and imaging to assess the condition properly.

Conventional intraoral dental radiography with multiple projections and angulations may give an adequate diagnostic yield of odontodysplasia. CBCT and conventional CT scans of the skull display detailed coronal, sagittal, and axial images with a larger field of view. The volume-rendered 3-dimensional images permit evaluation of the spatial relationship between teeth and maxillofacial structures, which may aid surgical treatment planning for odontodysplasia, especially for those that are deeply impacted or with secondary disease involving cysts. In our study, the three imaging modalities could identify small size, irregular crown and root shape, and odontodysplastic teeth malformation. However, when comparing the three cases that had two imaging modalities performed, intraoral dental radiographs, in contrast to CT or CBCT scan, failed to reveal detailed information of abnormal root morphology and orientation, pulpal and canal anatomy, and condition of the apex due to superimposition of adjacent teeth, or the odontodysplastic structures themselves. Thus, advanced imaging may contribute better to the localization of the odontodysplastic tooth and offer greater accuracy in planning surgical approaches.

Secondary diseases or lesions in odontodysplastic teeth were commonly seen in the dogs included in our study. The abnormal dental anatomy and irregular surface on the crown secondary to hypoplastic and hypocalcified enamel typically encourage plaque and calculus deposition and make the odontodysplastic tooth highly prone to periodontal disease. The affected tooth may have reduced fracture resistance secondary to its fragile structure. The invaginations extending from the enamel surface to the dentin of affected teeth have been observed (24), possibly allowing entry of bacteria, leading to pulpitis, pulpal necrosis, and formation of abscess (25, 26). Also, those secondary diseases were more frequently identified in RO-affected strategic teeth than non-strategic ones. This effect may be associated with our result: RO was significantly more commonly seen in strategic teeth than non-strategic teeth. Extraction was performed in 17/22 (77.3%) of RO cases in dogs; all RO-affected teeth with secondary diseases were extracted. The figure was comparable to human cases in which 78.6% underwent extraction (27). In humans, there is no standardized treatment guideline for clinical management of odontodysplasia; reported treatment options include extraction followed by prosthetic treatment, restoration, and orthodontic treatment for unerupted or partially erupted affected teeth (28). The treatment varies depending on the severity of the condition and the patient’s functional and aesthetic needs; often, it is a multidisciplinary approach (28). However, it was reported in the human literature that severe periapical infections and maxillofacial abscesses may occur later in all odontodysplastic teeth (29), and the formation of cysts and pressure-induced pain can be associated with the impacted odontodysplastic teeth (28). Regular follow-up to monitor the disease progress is essential in odontodysplasia cases with no extraction performed. In our study, follow-up data was only available in two of the five dogs with the diagnosed odontodysplastic tooth not being extracted, and one of them had the tooth assessed as severe periodontal disease at revisit, based on clinical and radiographic findings. Therefore, since dogs have minimal aesthetic needs, their unlikelihood of dental drifting after tooth loss, and potential loss to follow-up, veterinarians must be aware that conservative treatment for odontodysplastic teeth may not outweigh the risk of developing secondary disease. Of the 17 dogs that had extraction performed in our study, all the extraction sites healed uneventfully, except one that had a wound dehiscence 2 weeks post-operatively, which eventually was deemed healed. This success rate demonstrates that extraction is a viable and reasonable treatment for odontodysplasia in dogs. However, a small sample size limits firm conclusions.

The categorization of RO and GO adopted in this study concerns human medicine and takes the anatomical basis into account for the optimal presentation of our findings. The authors acknowledge that RO in humans may be a specific disease entity, but, to the authors’ knowledge, there is no consensus on the definition of a regional or generalized form of odontodyplasia in human literature in terms of the number of teeth or quadrants involved (1, 3–6). Nonetheless, the data obtained from this study revealed similarities in the epidemiological and clinical aspects of odontodysplasia (RO in particular) between humans and dogs. First, both humans and dogs share a female predilection for RO. The female-to-male ratio of RO was 1.37: 1 in humans (3), whereas in dogs, our study revealed a ratio of 2.14:1. The ratio goes higher to 5:1 in dogs with GO. Second, the maxilla was more commonly affected than the mandible in humans (3, 27) and dogs with RO. Only the maxilla was involved in 55.9% of human RO cases and 50% of dog RO cases; involvement of only the mandible was found in 34.2% of human cases and 31.8% of dog cases (3). Third, the signs and symptoms reported in human RO cases include failure of tooth eruption, swelling of the affected area, local pain, and periapical inflammation (3, 27); some of those were assessed, perceived, and evident in dog RO cases after thorough examination and diagnostics. Unlike humans, dogs cannot express symptoms; owners and veterinarians can only pick up signs to validate an investigation, thus justifying a comprehensive assessment.

Although local trauma has been discussed as a possible etiology for odontodysplasia or RO, similar dental abnormalities, for instance, failure of or partial eruption, abnormal tooth shape, enamel hypoplasia, and pulp necrosis, were reported in dogs with a history of mandibular fractures (14), local infection, teratogenic drug exposure, local circulatory disorders, Rh incompatibility, irradiation, neural damage, hyperpyrexia, metabolic disorder, nutritional deficiency, and activation of latent viruses in odontogenic epithelium with the presence of vascular nevus have been described as possible contributing factors (27). There is no consensus on explaining the occurrence of odontodysplasia in different locations and tooth types; the condition may be multifactorial causality (3) or idiopathic (29). The lack of reported history of local trauma in the cases described in this study supports the argument.

It is worth mentioning that historic distemper virus infection was reported in 66.7% of the dogs affected by GO in our study. The result is comparable to distemper virus infection, which is consistently reported as the most common cause of generalized enamel and dentin hypoplasia in dogs (30). The authors recently acknowledged a case (2-month-old male dog) with confirmed distemper infection using immunohistochemistry (IHC) on tissue samples, including the teeth, in which distemper viral inclusion bodies were identified in ameloblasts and odontoblasts (Supplementary Figure S1). The case was not included in the present study due to a lack of description in oral examination findings. However, it demonstrated the effect of the distemper virus on dental tissues. Besides viral infection, interestingly, diffuse change of the trabecular bone density noted in the CBCT scan of one of the GO cases is suggestive of possible resolving or resolved prior metabolic bone disease (e.g., nutritional secondary hyperparathyroidism), which may be associated with the occurrence of GO in the dog. However, the absence of systemic disease and other co-morbidities in many cases presented here suggests that no single etiological factor can readily explain the anomaly.

Retrospective analysis is an acceptable method to study the epidemiological and clinical data of rare disease entities like odontodysplasia (3). However, the study’s retrospective nature needs to be revised regarding the standardization of data collection and small samples submitted for histopathological analysis. Most of the diagnosis was made by comprehensive oral examination and imaging during anesthesia. Also, the owners were relied on to recall the dogs’ history, which may reflect a partial history, such as historic distemper infection or another incidence prior to acquiring the dog. Hence, incomplete data or bias may be present.

This study summarizes the data on the prevalence, clinical presentation, diagnosis, and treatment of odontodysplasia in dogs with unknown trauma history and the secondary diseases or lesions associated with this rare anomaly. Accurate history taking, a thorough oral examination, and appropriate imaging diagnostics are necessary to identify this condition and associated pathology and formulate the most appropriate treatment plan for our patients. The etiology of the anomaly is yet to be determined, but the condition may be of multifactorial causality.

## Data availability statement

The original contributions presented in the study are included in the article/Supplementary material, further inquiries can be directed to the corresponding author.

## Ethics statement

Ethical review and approval were not required for the animal study because the study is retrospective in nature, hence, it is exempt from the Institutional Animal Care and Use Committee (IACUC) requirements. The standard written informed consent was required for all procedures performed at the William R. Pritchard Veterinary Medical Teaching Hospital of the University of California, Davis and was obtained from the owners.

## Author contributions

CCSK: Conceptualization, Formal analysis, Visualization, Writing – review & editing, Data curation, Investigation, Writing – original draft. SG: Formal analysis, Visualization, Writing – review & editing, Methodology. NV: Formal analysis, Methodology, Visualization, Writing – review & editing, Data curation, Investigation, Resources. BA: Writing – review & editing, Conceptualization. MS-R: Conceptualization, Writing – review & editing, Formal analysis, Methodology, Resources, Supervision, Visualization.
