# Supplementary material for: Clinicial-pathologic correlations of non-trauma related Odontodysplasia in 28 dogs: 2013-2023
Source: Front Vet Sci. 2024 Jul 8;11:1424784. doi: 10.3389/fvets.2024.1424784 (PMC11260788; doi:10.3389/fvets.2024.1424784)
Supplement: Supplementary file 1 [file Data_Sheet_1.docx]

| **Clinical data of Regional Odontodysplasia (RO)** | **Number of RO-affected Teeth (%)** |
| --- | --- |
| Strategic tooth | 14 (34.1) |
| Non-strategic tooth | 27 (65.9) |
|  | |
| *Specific tooth in Right Maxillary quadrant* | |
| First incisor tooth | 0 (0) |
| Second incisor tooth | 2 (4.9) |
| Third incisor tooth | 1 (2.4) |
| Canine tooth | 3 (7.3) |
| First premolar tooth | 2 (4.9) |
| Second premolar tooth | 1 (2.4) |
| Third premolar tooth | 0 (0) |
| Fourth premolar tooth | 2 (4.9) |
| First molar tooth | 2 (4.9) |
| Second molar tooth | 2 (4.9) |
|  | |
| *Specific tooth in Left Maxillary quadrant* | |
| First incisor tooth | 1 (2.4) |
| Second incisor tooth | 0 (0) |
| Third incisor tooth | 2 (4.9) |
| Canine tooth | 3 (7.3) |
| First premolar tooth | 1 (2.4) |
| Second premolar tooth | 0 (0) |
| Third premolar tooth | 2 (4.9) |
| Fourth premolar tooth | 1 (2.4) |
| First molar tooth | 0 (0) |
| Second molar tooth | 0 (0) |
|  | |
| *Specific tooth in Left Mandibular quadrant* | |
| First incisor tooth | 1 (2.4) |
| Second incisor tooth | 0 (0) |
| Third incisor tooth | 1 (2.4) |
| Canine tooth | 0 (0) |
| First premolar tooth | 1 (2.4) |
| Second premolar tooth | 1 (2.4) |
| Third premolar tooth | 0 (0) |
| Fourth premolar tooth | 1 (2.4) |
| First molar tooth | 2 (4.9) |
| Second molar tooth | 1 (2.4) |
| Third molar tooth | 2 (4.9) |
|  | |
| *Specific tooth in Right Mandibular quadrant* | |
| First incisor tooth | 0 (0) |
| Second incisor tooth | 0 (0) |
| Third incisor tooth | 0 (0) |
| Canine tooth | 0 (0) |
| First premolar tooth | 2 (4.9) |
| Second premolar tooth | 0 (0) |
| Third premolar tooth | 0 (0) |
| Fourth premolar tooth | 0 (0) |
| First molar tooth | 3 (7.3) |
| Second molar tooth | 1 (2.4) |
| Third molar tooth | 0 (0) |

Supplementary Table 1. Clinical data of teeth affected by Regional Odontodysplasia (RO)

*Strategic tooth refers to any of the following: maxillary and mandibular canine tooth, maxillary fourth premolar tooth and mandibular first molar tooth


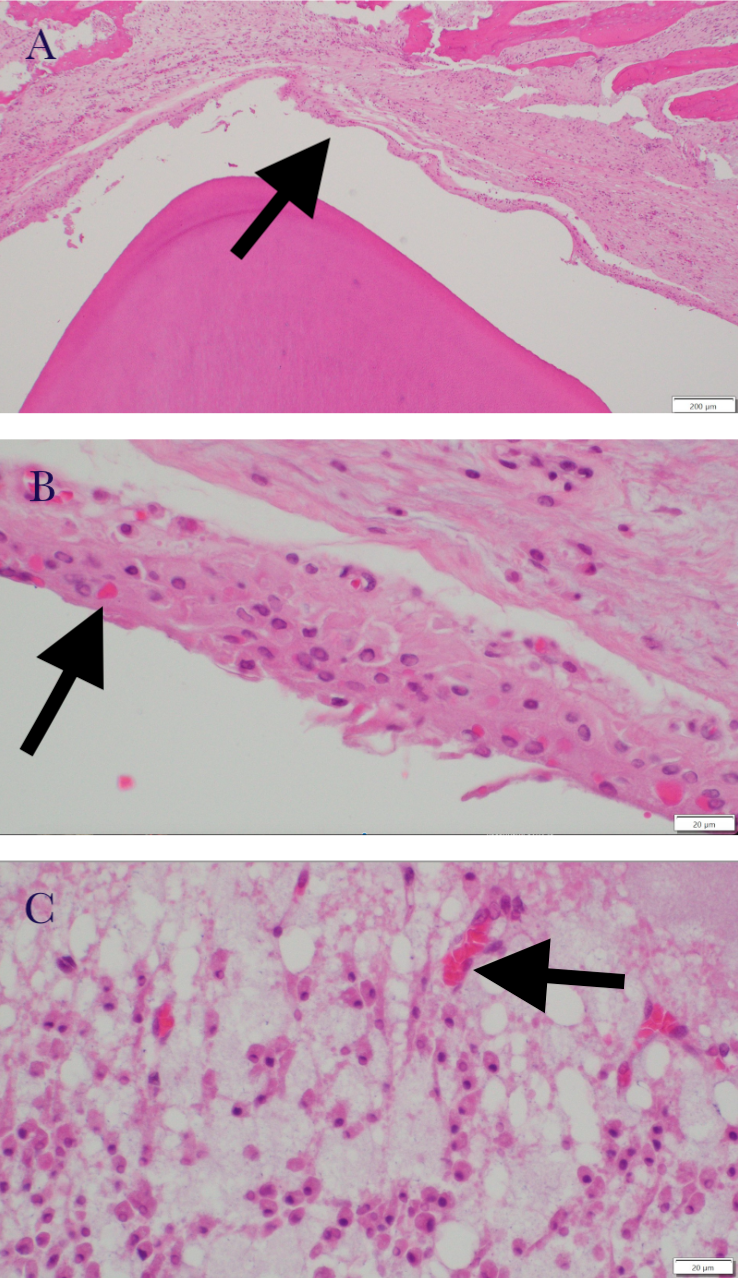


Supplementary Figure 1. Histology slides (HE stain) of the unerupted permanent tooth of a 2-month-old dog with distemper infection. (A) Abnormal ameloblast epithelium (arrow). (B) Ameloblasts with viral intranuclear inclusions (arrow). (C) Odontoblasts with viral inclusions (arrow).
